# Analyzing the facial nerve at Zuker’s point using geometric morphometrics: a cadaveric study

**DOI:** 10.1186/s40902-025-00481-w

**Published:** 2025-09-29

**Authors:** Aaron W. Beger, Priyanka Shah, Tori Womble, Yash Desai, John Massie, Prutha Patel, Brandon Raquet, Jonathan A. Millard

**Affiliations:** 1 Department of Biomedical Sciences, Edward Via College of Osteopathic Medicine, Blacksburg, United States; 2 Edward Via College of Osteopathic Medicine, Blacksburg, United States

**Keywords:** Facial nerve, Zuker’s point, Geometric morphometrics, Rhytidectomy, Cranial nerve VII, Extracranial, Zygomatic branch, Zygomaticus major

## Abstract

**Background:**

Surface landmarks such as Zuker’s point can help localize branches of the facial nerve as a supplement to, or in the absence of intraoperative neuromonitoring. Zuker’s point is a previously described landmark located half the distance between the root of the auricular helix and labial commissure with demonstrated efficacy in localizing facial nerve branches. However, prior studies are restricted by two dimensional descriptions based on discrete, linear measurements, and description of the influence of sex, laterality, or face shape is lacking. Geometric morphometric techniques offer a sophisticated approach capable of producing a three-dimensional description of the location of the facial nerve relative to Zuker’s point, while discerning the influence of sex, laterality, and face shape.

**Methods:**

Facial nerve at Zuker’s point was analyzed in 82 cadaveric hemifaces. Three-dimensional coordinate data for the root of the auricular helix, labial commissure, Zuker’s point, and the facial nerve at Zuker’s point were captured using a MicroScribe digitizer. Mean landmark configuration and principal component analysis were used to describe the position of the facial nerve relative to Zuker’s point, as well as the influence of sex, laterality, and face shape.

**Results:**

Facial nerve systematically deviated towards the 1 o’clock position (if viewed from the lateral position on the right side), irrespective of sex or laterality. The accuracy of Zuker’s point was enhanced on those with a longer, rounder face, while in shorter and slimmer faces the nerve takes a more superior and superficial position relative to Zuker’s point.

**Conclusions:**

Facial nerve branches can reliably be found at Zuker’s point, regardless of sex or laterality, though the nerve does systematically deviate from Zuker’s point toward the 1 o’clock position (if viewed on the right side). The position of the facial nerve relative to Zuker’s point is influenced by face shape, but not sex or laterality. These results can be used to better localize the facial nerve and improve patient outcomes.

**Supplementary Information:**

The online version contains supplementary material available at 10.1186/s40902-025-00481-w.

## Background

Damage to the extratemporal motor branches of the facial nerve (FN) disrupts innervation to mimetic muscles and can lead to debilitating physical and psychosocial sequelae [1–4]. Iatrogenic injury should be suspected in cases of unexpected postoperative facial weakness, representing a potentially preventable etiology that has been estimated to account for 5–7% of all FN injuries [5, 6], with potentially higher risk depending on procedure and approach [7–9]. Procedures that leave the FN most vulnerable to iatrogenic injury include temporomandibular joint reconstruction and repair, parotidectomy, removal of lesions of the head and neck including facial neuromas, interventions following facial trauma, and cosmetic procedures including rhytidectomy, highlighting its relevance in a wide range of surgical specialties and subspecialties [5, 10–12]. Iatrogenic FN injury has also been cited as the most commonly litigated cranial nerve injury [13], further emphasizing the call for preventative measures.

Prevention of iatrogenic FN injury is complicated by its plexiform arrangement which exhibits a high degree of variation in its patterning and course [10]. Intraoperative neuromonitoring can assist with FN mapping and has been shown to prevent short-term neurological deficits during parotidectomy, though its utility in preventing permanent FN damage is inconclusive [14–16]. Neuromonitoring also relies on specialized training that was formally offered in only 61% of otolaryngology residencies as of 2018 [17], and may require heavy modulation in patients with pseudocholinesterase deficiency or those needing high doses of non-depolarizing anesthetic agents [18, 19].

Reliable surface landmarks have therefore been established to localize FN in the absence of, or as a supplement to, intraoperative neuromonitoring. One of which is Zuker’s point (ZP), located midway between the root of the auricular helix and labial commissure, which has demonstrated utility in localizing the FN branch responsible for innervating the zygomaticus major muscle. The first published description of ZP was by Dorafshar et al. in 2013 in which the FN was found to be an average of 2.31 mm (range: 0-6 mm) from subcutaneous ink injected at ZP in 18 cadaveric hemifaces, with data on directionality limited to FN being either superior to (6/18; 33%), inferior to (5/18, 28%), or at ZP (7/18; 39%) [20]. Follow-up dissection studies have found FN branches within 5.0 mm of ZP in the majority of subjects [21, 22]; however, the use of discrete, linear measurements restricts three-dimensional understanding, and lack of investigation around sex and laterality differences further limits the clinical utility of these results.

Geometric morphometrics (GM) offers a more sophisticated methodology capable of revealing the positioning of FN at ZP in three dimensions and a more novel approach for scrutinizing the surgical anatomy of FN at ZP. GM employs a landmark-based approach to explaining variation in shape through the capture and analysis of Cartesian (e.g. x, y, z) coordinates, with an emerging role in craniofacial surgery [23]. Further, it allows for quantitative description of sex and laterality differences. Similar methods have been used to scrutinize Pitanguy’s line, a surface landmark for approximating the location of the frontotemporal branch of FN [24].

Therefore, the aim of this study was to describe the location of FN in relation to ZP using GM in a sample of adult whole-body donors. GM techniques were used to expose trends in the relationship between FN and ZP, while principal component analysis was used to explore patterns in the landmark configuration to determine the influence on facial arrangement on FN position. These results can be used to delineate a critical zone during facial surgery to help prevent iatrogenic FN injury, while also limiting dissection area and operative time when searching for FN, with the ultimate aim of improving patient outcomes.

## Methods

The aim of this study was to describe the three-dimensional positioning of the extraparotid FN in relation to ZP using a postmortem sample of adult whole body donors. GM techniques allowed for higher-dimensional resolution for the description of the FN’s position in relation to ZP and surrounding landmarks.

### Subjects

A total of 57 adult whole body donors willed to Edward Via College of Osteopathic Medicine via the Virginia State Anatomical Program (n = 48) or University of Massachusetts Chan Medical School anatomical gift program (n = 9) were recruited via convenience sampling. Inclusion criteria included voluntary informed consent provided by the donor for their tissue to be used for research and academic purposes. Exclusion criteria included trauma, congenital malformations, or surgical history of the maxillofacial region either made evident at the time of dissection or listed in the provided donor paperwork, as well as damage to, or inability to locate the FN during the dissection protocol.

### Dissection

**Fig. 1:**
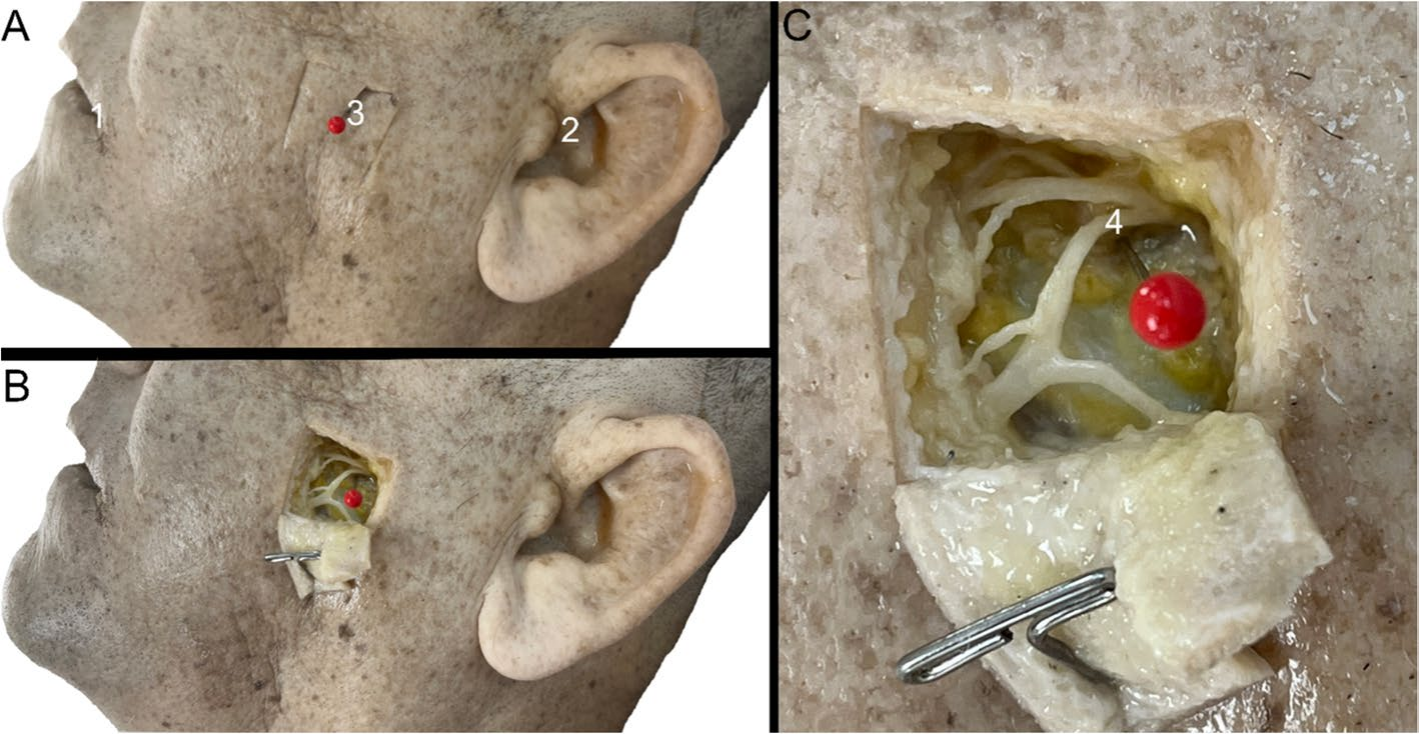
Overview of dissection protocol. **A** The distance between the labial commissure (1) and root of the auricular helix (2) was halved to identify the location of ZP (3), which was marked with a pin. **B** The square inch of skin around the pin was cut and reflected, and the subcutaneous tissue was removed to reveal branches of FN. **C** Zoomed-in view of dissected area seen in (**B**) to depict where FN was located nearest to the pin (4)

ZP was localized bilaterally by first measuring the distance from the root of the auricular helix to the labial commissure with string, then piercing the skin with a pin at the halfway point (Fig. 1A). The square inch of skin surrounding the pin was cut and reflected, and the superficial and deep fascial layers were excised via sharp dissection to reveal the FN branch nearest the pin (Fig. 1B, C). Dissection was limited to a discrete area to maintain the donor’s primary usage in our medical curriculum as an educational resource via student dissection. Therefore, expansive exploratory dissection to identify the specific FN branch at ZP was not permitted by the researchers. Dissection protocol was conducted unilaterally on 23 donors and bilaterally on 34 donors, for a total of 91 sides.

### Data Collection

A calibrated MicroScribe desktop digitizer (*i* + series; revware.net) was used to bilaterally capture the three-dimensional coordinate data for the four landmarks of interest: labial commissure, root of the auricular helix, ZP, and the branch of FN traveling nearest to ZP. Landmark data was collected by three independent investigators (JM, PS, and TW) and the raw coordinate data was imported to a Microsoft Excel spreadsheet (version 2501).

### Statistical Analysis

Raw coordinate data was uploaded to RStudio for geometric morphometric analyses using the R package *geomorph* (v. 4.0.10) [25, 26]. Procrustes superimposition was used to align homologous landmarks by translating, scaling, and rotating the landmark configuration, and reveal the three-dimensional mean of the landmark configuration. This represents the most common statistical superimposition method in geometric morphometrics [27]. Variations in the Procrustes aligned coordinates were explored using a principal component analysis (PCA). PCA is used to reduce dimensionality and expose patterns in the landmark configuration that explain the most variation in the dataset. Differences in the landmark configurations of males versus females and left versus right sides were explored by comparing principal component (PC) scores via Mann–Whitney U test (α = 0.05). Procrustes distance (PD) was calculated by summing the squared difference in PC scores for homologous landmarks. Landmark configurations with PD 2.25 times greater than or equal to the mean PD were considered statistical outliers and removed (Table 1).

**Table 1:** Demographic information of subjects. Statistical outliers were identified as sides with landmark configurations that displayed a PD 2.25 times greater than or equal to the sample mean PD (sample mean PD = 0.29; outlier cutoff PD = 0.6525)

	***n* (sides)**	**Age (years)** $\bar{\text{X}}$ **± SD**	**Sex** **M:F**
Statistical outliers removed from sample	9	87.66 ± 8.07	2:7
Final sample included in study	82	82.23 ± 11.33	42:40

*F* female, *M* male, *PD* Procrustes distance, *SD* standard deviation, $\bar{\text{X}}$ mean

### Error Study

Sophistication and repeatability of the landmarking protocol was established via an error study, aligning with GM standards. Landmarking was independently replicated on a subset of the sample (67/91 sides; 74%) by independent investigators (JM and TW) at two separate times. Repeatability of the landmarking protocol was determined by comparing PD of the replicated dataset via Procrustes ANOVA as established by Goodall [28] and supported by Ito et al [29]. Failing to reject the null hypothesis of the Procrustes ANOVA (*p* > 0.05) would indicate that the identity of the landmarker did not have significant influence on the variation of the dataset, allowing the coordinate data captured by the three investigators (JM, PS, and TW) to be combined.

## Results

A total of nine landmark configurations were removed as statistical outliers as they yielded a PD 2.25 times greater than or equal to the sample mean PD (sample mean PD = 0.29; outlier cutoff PD = 0.6525) (Fig. S1). Inspection of the outlier landmark configurations revealed two general patterns. First, the majority of outliers were found to be anatomically infeasible and reflect errors in data capture (e.g. ZP not being halfway between the labial commissure and root of the auricular helix, or FN being superficial to ZP) (Fig. S2). Second, a minority of outliers appeared anatomically feasible but instead likely represent anomalous or extreme anatomical variations (e.g. ZP being far lateral, indicating especially rounded cheeks, paired with a particularly deep FN) (Fig. S3).

The final sample included in the analysis was therefore 82 sides. Demographic information of outlier and included subjects is provided in Table 1.

### Error Study

Landmarking was replicated on a subset of the sample by two investigators (JM and TW) as a way to establish sophistication and repeatability of the landmarking protocol. Testing the PD of the replicated datasets via Procrustes ANOVA did not reveal a significant difference (*p* = 0.445, Table 2), indicating independent landmarkers were consistent and had a reasonable amount of agreement in identifying landmarks. This allowed for combination of the datasets collected by the three landmarkers (JM, PS, and TW) for subsequent analyses.

**Table 2:** Results of Procrustes ANOVA for error study, designed to test for differences in the data collected by two independent investigators (α = 0.05)

Factor	Df	SS	MS	R^2^	F-value	Z-score	*p*-value
Landmarker	1	0.1098	0.1098	0.0076	0.9982	0.1669	0.445
Residuals	131	14.3759	0.10974	0.99238	-	-	-
Total	132	14.4864	-	-	-	-	-

*Df* degrees of freedom, *MS* mean squares, *SS* sum of squares

### Left vs. Right

Laterality of the landmark configuration was scrutinized by comparing the PD of left and right sides via Procrustes ANOVA. Results revealed there to be no significant difference between the left and right sides (*p* = 0.361, Table 3). Therefore, left and right sides were combined for subsequent analyses by multiplying the Procrustes aligned coordinates in the Y plane by −1, mirroring all left landmarks to appear as right.

**Table 3:** Results of Procrustes ANOVA for differences in sex and laterality (α = 0.05)

Factor	Df	SS	MS	R^2^	F-value	Z-score	*p*-value
Sex (M/F)	1	0.0662	0.066199	0.00863	0.7081	−0.26420	0.603
Side (L/R)	1	0.1036	0.103576	0.01350	1.1080	0.36539	0.361
Sex × side	1	0.2135	0.213495	0.02782	2.2838	1.68465	0.042*
Residuals	78	7.2917	0.093484	0.95006	-	-	-
Total	81	7.6750	-	-	-	-	-

*Df* degrees of freedom, *L/R* left/right, *M/F* male/female, *MS* mean squares, *SS* sum of squares

^*^ *p* < 0.05

### Male vs. Female

Variation between sexes was explored by comparing PD in males versus females via Procrustes ANOVA. Results indicated no significant difference in the landmark configuration between males and females (*p* = 0.603, Table 3), allowing these groups to be combined for subsequent analyses.

To test the hypothesis that males have an overall larger landmark configuration, centroid sizes (CS) were compared between sexes (Table 4). CS reintroduces size to the configurations, an element that was initially removed via scaling of homologous landmarks as part of the Procrustes superimposition. Though the CS of males ($\bar{\text{X}}$ = 1289.33) was found to be slightly larger than females ($\bar{\text{X}}$ = 1166.74) these groups were not found to be significantly different based on Mann Whitney U test (*p* = 0.149).

**Table 4:** Comparison of centroid size in males (M) versus females (F) via Mann Whitney U test (α = 0.05)

Sex	*n*	Centroid size ($\bar{\text{X}}$)	*p*-value
M	40	1289.33	0.149
F	42	1166.74	

### Mean Landmark Configuration

**Fig. 2:**
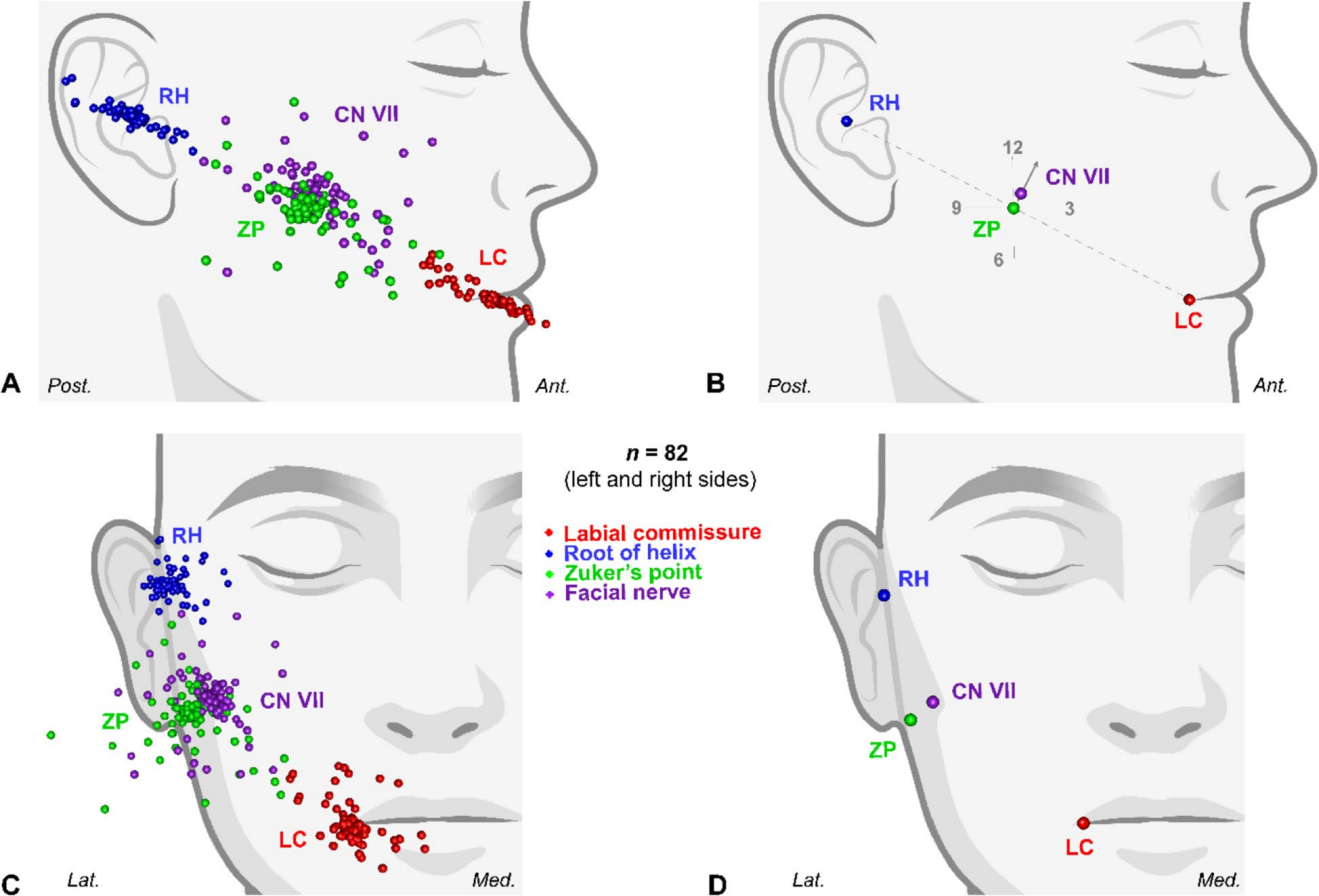
Results from 82 sides visualized in lateral (A,B) and anterior (C,D) views, depicting Procrustes aligned coordinates (A,C) and mean landmark configuration (B,D) (figure created using Biorender.com). *CN VII, facial nerve at Zuker’s point; LC, labial commissure; RH, root of auricular helix; ZP, Zuker’s point*

Following the removal of statistical outliers, the Procrustes aligned coordinates of the combined and mirrored 82 sides were used to derive the mean landmark configuration (Fig. 2).

Although ZP was relatively reliable in localizing FN, the average location of the nerve was found to systematically deviate towards the 1 o’clock position in relation to ZP when viewed from a straight lateral perspective on the right side, or 11 o’clock if viewed from the left side (Fig. 2A, B). When viewed anteriorly, the landmark configuration confirmed ZP to be midway between labial commissure and root of the helix, and FN to be deep to ZP, as predicted (Fig. 2C, D).

### Principal Component Analysis (PCA)

PCA was used to expose patterns, or “principal components” (PC), in the landmark configuration that explain the most variation in the dataset. PCA revealed a total of five PCs, with percentage of variation explained by each summarized in Table 5. PC1 and PC2 collectively accounted for over 58% of the variation in the sample, with PC1 alone accounting for over 33%. Therefore, emphasis was placed on the biological interpretation of PC1 as it represented the landmark configuration that accounted for the greatest variation in the sample.

**Table 5:** Results of Principal Component (PC) Analysis

PC	Eigenvalue	Variance explained (%)	Cumulative variance (%)
1	0.02633427	33.740	33.740
2	0.01912687	24.506	58.245
3	0.01535423	19.672	77.917
4	0.00881789	11.298	89.215
5	0.00841782	10.785	100

**Fig. 3:**
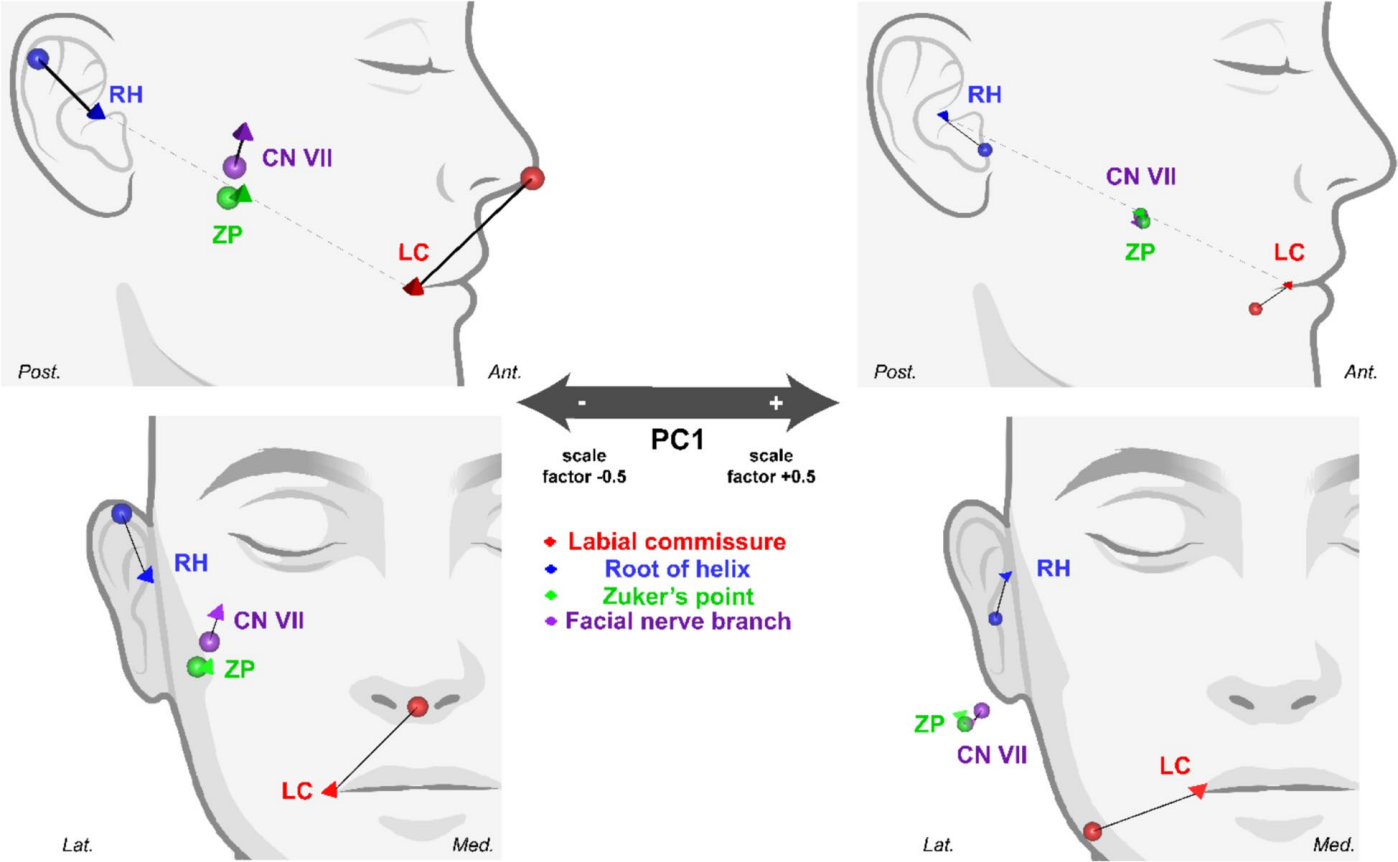
Scaling of the landmark configuration along the first principal component (PC1). The mean landmark location is represented by the corresponding colored sphere, with influence of PC1 represented by the pyramid. *CN VII, facial nerve at Zuker’s point; LC, labial commissure; RH, root of auricular helix; ZP, Zuker’s point*

Positive and negative scaling of PC1 depicted how the landmark configuration was altered along this principal component, revealing its biological significance (Fig. 3). Negative scaling of PC1 by a factor of −0.5 demonstrated a shorter distance between the labial commissure and root of the helix. As the distance between the lips and ear decreased along PC1, the FN migrated superficially and away from ZP in a superior direction. Positive scaling of PC1 by a factor of + 0.5 corresponded with increased distance between the ear and lips. As the distance between the ear and lip increased along PC1, the FN was found to be closer to ZP, meaning ZP had greater predictive value in localizing FN in those with this facial arrangement.

To discern whether this pattern was significantly influenced by sex, male and female PC scores of PC1-3 were compared with one another via Mann Whitney U test. Results indicated that the variations in the landmark configuration along the first three PCs were not significantly influenced by sex (Table 6). Therefore, the positive and negative scaling of PC1 as previously described, as well as the variations represented by PC2 and PC3, can be predicted regardless of sex.

**Table 6:** Comparison of principal component (PC) scores (PC1-PC3) between males and females via Mann Whitney U test (α = 0.05)

PC	*p*-value
1	0.930
2	0.923
3	0.457

## Discussion

Understanding FN anatomy is imperative for avoiding iatrogenic injury during surgical interventions of the face. Surface landmarks like ZP can facilitate efficient localization of FN, though the influence of different variables across various populations should be continually studied if their utility is to be optimized. This study employed GM techniques to produce a sophisticated three-dimensional description of the relationship between FN and ZP within a cadaveric sample, permitting novel exploration of the influence of sex, laterality, and face shape. These results can improve patient outcomes by decreasing operative time through more efficient localization of FN during grafting procedures or delineating a critical zone to prevent nerve injury while minimizing the dissection field [20].

The specific FN branch found at ZP has most often been found to be a tertiary branch derived from the frontozygomatic/upper division [21] and typically carries over 900 axons, though this may be influenced by the type of FN arborization pattern [22]. This nerve is responsible for innervating mimetic muscles of the midface, including the zygomaticus major muscle, an important facilitator of joyful expression [30]. As such, it is often a target in facial reanimation procedures via crossface nerve grafting with the aim of establishing spontaneous smiling in patients with unilateral FN damage [31]. Employing ZP as a supplement to neuromonitoring could expedite the identification of FN in such procedures, helping to minimize operative time. We found FN to be near ZP in 82 hemifaces, supporting the landmark’s relative reliability in localizing FN. However, the average location of FN systematically deviated towards the 1 o’clock position in relation to ZP when viewed from a straight lateral perspective on the right side (11 o’clock if viewed from the left), irrespective of sex or laterality. While this aligns with the finding of FN being found superior to, or at ZP in 72% of hemifaces reported in the original study [20], it adds a more refined element of directionality. This more critical analysis of ZP can also be used alongside other anatomical landmarks for frontotemporal [24, 32] and marginal mandibular [33, 34] FN branches in the delineation of critical zones to help prevent nerve injury during surgical interventions of the face such as rhytidectomy.

An important element of ZP is that it is based on relative measurements of fixed anatomical landmarks (i.e. half the distance between the root of the auricular helix and labial commissure), allowing it to feasibly be applied to all patients regardless of age or face shape [20]. This is in contrast to other reference landmarks that rely on discrete distance measurements for approximating FN, such as Pitanguy’s line for approximating the course of the frontotemporal branch of FN (i.e. line drawn from 0.5 cm below the tragus to a point 1.5 cm superior to the lateral aspect of the eyebrow) [32]. However, the employment of PCA herein revealed that accuracy of ZP may in fact be influenced by a patient’s face shape (Fig. 3). ZP and FN were more closely approximated as the ear and lips migrated away from one another, suggesting ZP may have greater predictive value in localizing FN in those with this facial arrangement (i.e. longer and rounder faces). Meanwhile, individuals with shorter, slimmer faces, marked by decreased distance between the ear and mouth, had FN deviated more superficially and away from ZP in a superior direction. Therefore, the predictive value of ZP in localizing FN may be less accurate in those with shorter, slimmer faces, suggesting some degree of modulation may be necessary for individuals with this facial type. These findings contradict initial reports that promoted the accuracy of ZP irrespective of patient face size [20],and provide novel insight into how the accuracy of ZP may be governed by differences in facial arrangement. These differences may in part be due to the variable volume of deep and superficial fascial layers observed in the anterior midcheek [35], particularly the former as it is the layer within which FN travels in the region of ZP [36]. Increased fascial volume, manifesting as rounder cheeks, could feasibly influence the positioning of FN in relation to surface landmarks, though future studies would be needed to determine if any such relationship exists. Importantly, our study only examined adult subjects and merely offers preliminary insight into how face shape and size can influence FN positioning. This relationship could be further explored in future GM studies that incorporate subjects of varying ages and ancestral backgrounds to better elucidate this relationship.

While the use of GM in this cadaveric study offered novel insight into the relationship between ZP and FN, interpretation of results should be considered in the context of a variety of limiting factors. The nature of the study required FN to be revealed via sharp dissection. Though care was taken to minimize the disturbance of FN from its normal in situ position, it is feasible that mobilization of the nerve occurred during this process, disturbing the relative position of FN with ZP and influencing the results. Further, it is not known how tissue changes that occurred via the postmortem and embalming processes may have influenced our findings. Being able to ascertain the specific FN branch at ZP would also strengthen future studies. Employing a quantitative outlier threshold allowed us to objectively establish our final sample; however, in doing so we likely removed not only outliers due to error in data capture, but also individuals that fell outside of the normative range. Our model therefore only represents a typical morphometric profile and is not reflective of the anatomical extremes that may be encountered in clinical practice. With this in mind, surgeons should be aware of anatomical variations that may potentially influence the relationship between FN and ZP, which were not represented in our model. Finally, ancestral and age data were not included in the analysis and extrapolation of our results for individuals of varying ages (including pediatric populations) and ancestral backgrounds should be tempered. Future GM studies should include individuals of varying ages and ancestral backgrounds to determine the accuracy of ZP based on these variables. Such studies could also aim to establish clinically salient proxies of facial roundness and length in relation to FN at ZP. Doing so could provide more refined insight into the influence of face shape and size on FN positioning at ZP and promote its accessibility and utility as a clinical tool.

## Conclusions

ZP is a surface landmark located halfway between the root of the auricular helix and labial commissure and is relatively reliable in localizing the branch of FN that the zygomaticus major muscle. GM was used to establish a typical morphometric profile of the relationship between ZP and FN, revealing FN to systematically deviate from ZP in the 1 o’clock direction when viewed from a straight lateral perspective on the right side (11 o’clock if viewed from the left), and this was irrespective of sex. The accuracy of ZP in localizing FN is enhanced in those with longer and rounder faces, while in shorter and slimmer faces FN takes a more superior and superficial position. Not represented in this model were anatomical extremes, which must be considered in clinical practice as a potential influence on the position of FN at ZP. These results can be used to improve patient outcomes by preventing iatrogenic FN injury while enhancing surgical efficiency when localizing FN in grafting procedures.

## Supplementary Information

Supplementary Material 1.

Supplementary Material 2.

Supplementary Material 3.

Supplementary Material 4.

Supplementary Material 5.

## Data Availability

The coordinate dataset generated during the current study are available in the Figshare repository, 10.6084/m9.figshare.29316047.v1.

## Abbreviations

GM: Geometric morphometrics
FN: Facial nerve
PC: Principal component
PCA: Principal component analysis
PD: Procrustes distance
ZP: Zuker’s point
