# Supplementary figures and images for: Analyzing the facial nerve at Zuker’s point using geometric morphometrics: a cadaveric study
Source: Maxillofac Plast Reconstr Surg. 2025 Sep 29;47(1):25. doi: 10.1186/s40902-025-00481-w (PMC12480145; doi:10.1186/s40902-025-00481-w)

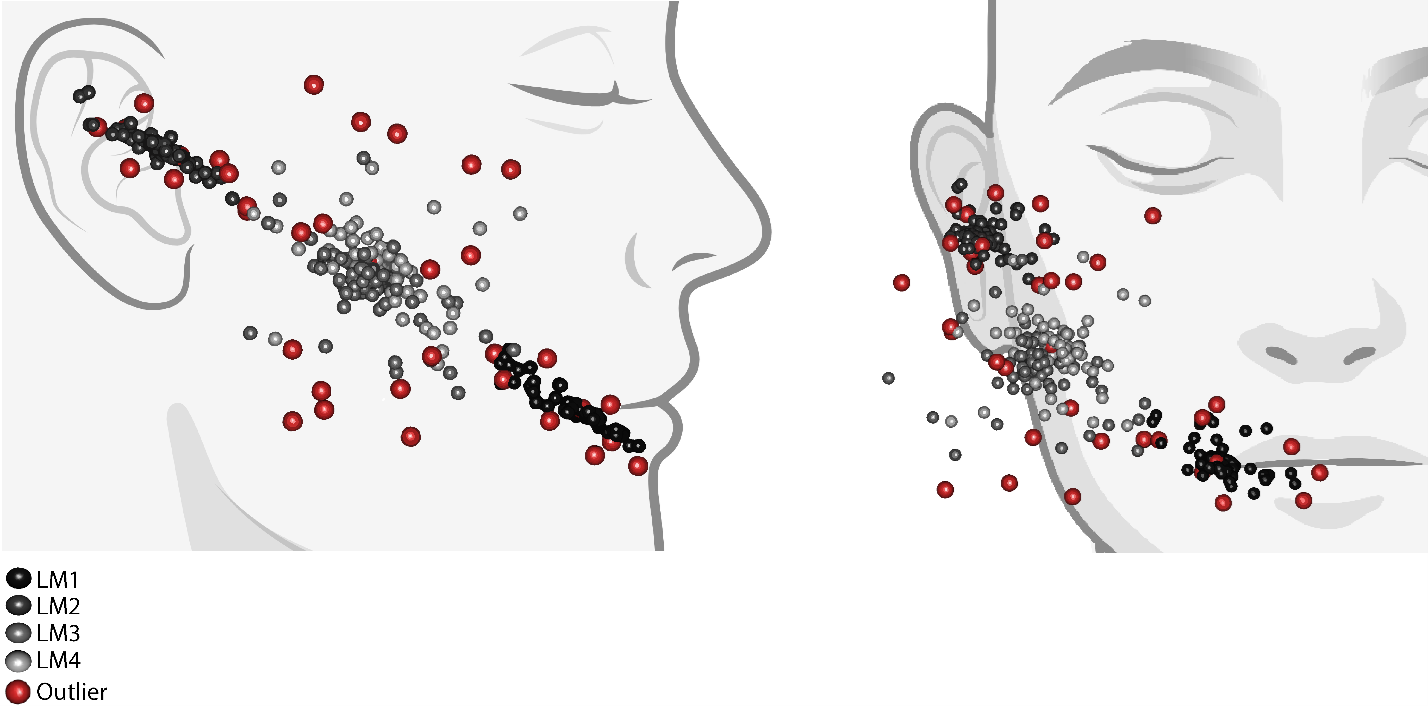

Supplement: Supplementary file 1 — Supplementary Material 1. [file 40902_2025_481_MOESM1_ESM.png]

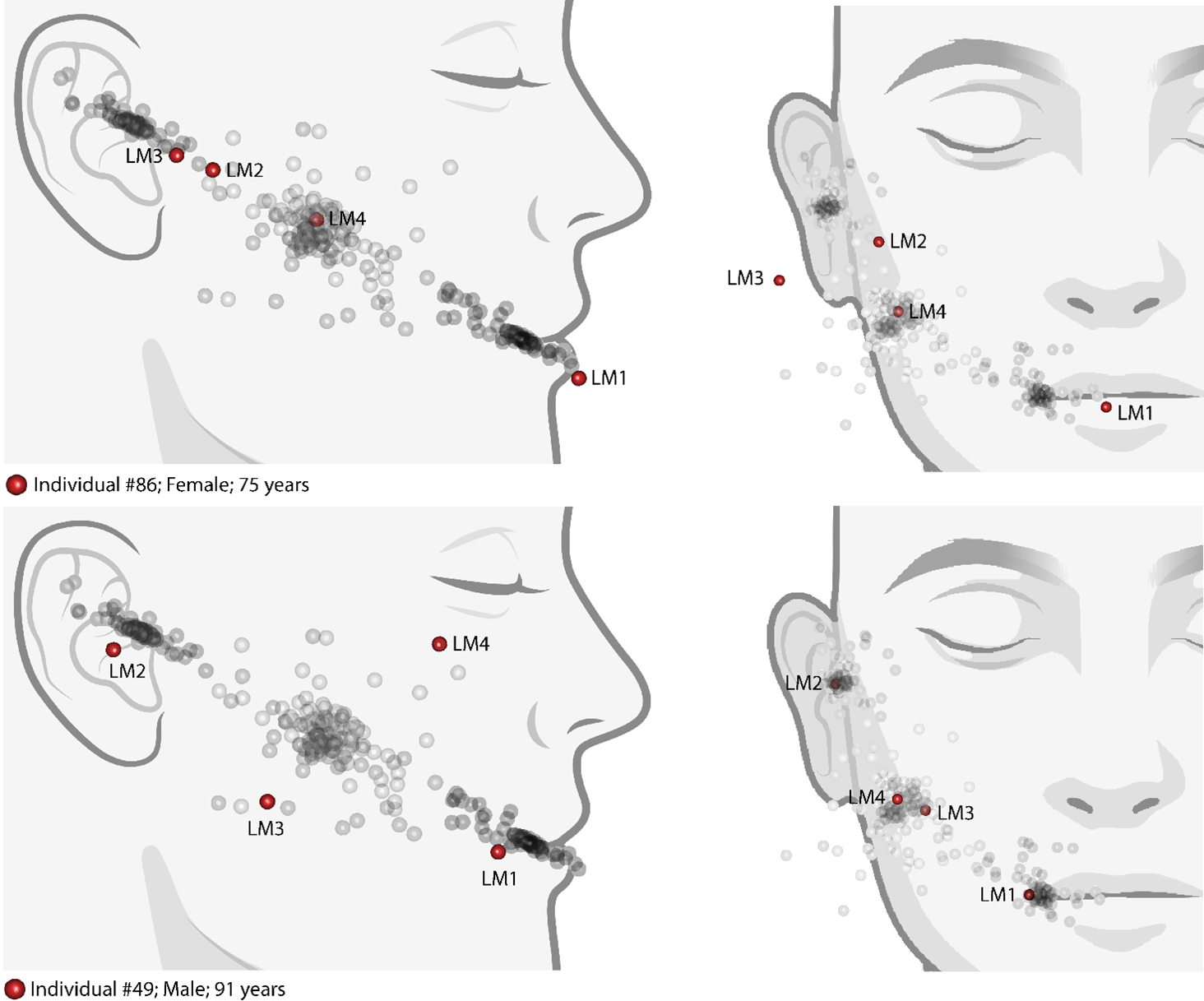

Supplement: Supplementary file 2 — Supplementary Material 2. [file 40902_2025_481_MOESM2_ESM.png]

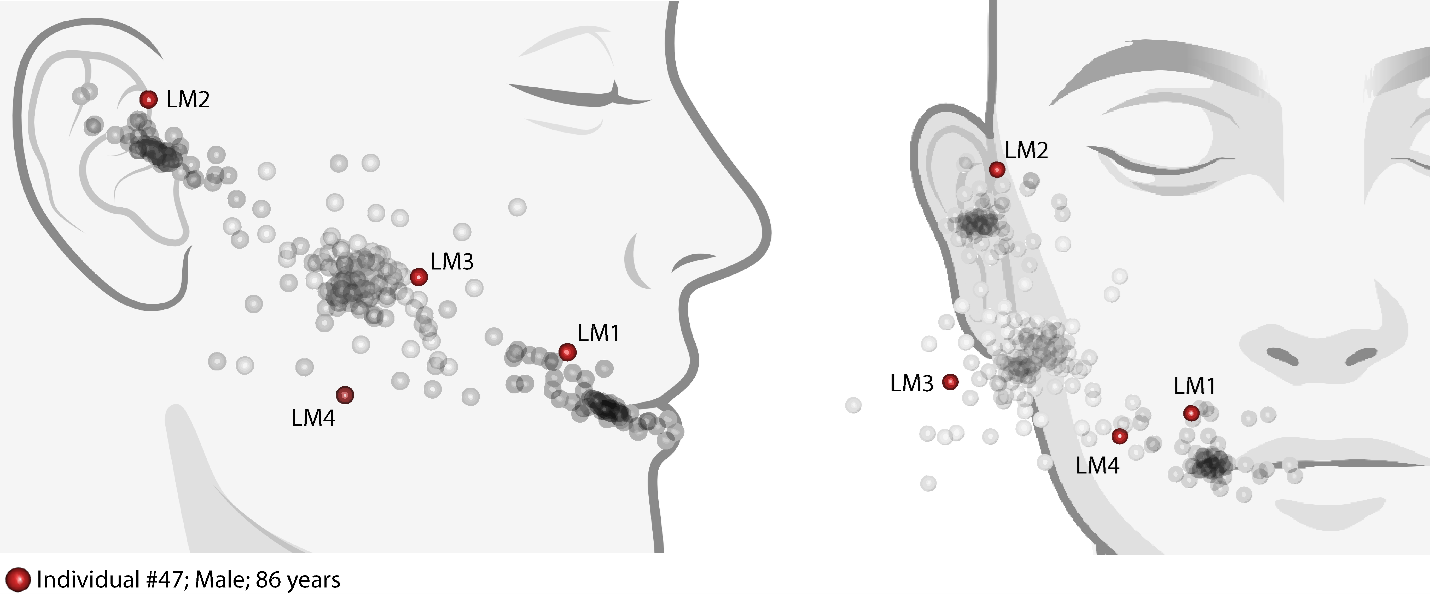

Supplement: Supplementary file 3 — Supplementary Material 3. [file 40902_2025_481_MOESM3_ESM.png]
